# The Biochemical Effects of Silver Nanoparticles and *Spirulina* Extract on Experimentally Induced Prostatic Cancer in Rats

**DOI:** 10.1007/s12011-022-03298-0

**Published:** 2022-06-11

**Authors:** Afaf D. Abd El-Magid, Omnia M. AbdEl-Hamid, M. A. Younes

**Affiliations:** Department of Biochemistry, Faculty of Veterinary Medicine, Benha University, Mushtuhur, Touch, Al Qalyubia Governorate, Benha, Egypt

**Keywords:** Prostatic cancer, Prostatic intraepithelial neoplasia, Silver nano-particles, *Spirulina* extract, Bicalutamide

## Abstract

Prostate cancer (PCa) is the most diagnosed cancer in 112 countries and the second leading cause of death in men in 48 countries. We studied the outstanding agents silver nanoparticles (AgNPs) and *Spirulina* algae (Sp) for the management of PCa once as monotherapy or last as a combination. PCa in rats was induced using bicalutamide (Casodex®) and testosterone, followed by (7, 12-dimethylbenz[a]anthracene). Then, testosterone was injected s.c. for 3 months. Rats were divided into six groups, with 12 rats in each group. Group I was assigned as the control (co), group II as the PCa model, group III treated with AgNPs, group IV treated with *Spirulina* extract, group V treated with a combination of AgNPs plus *Spirulina*, and group VI treated with bicalutamide. The results show that AgNPs could normalize IL-6 levels and could overcome the hormonal disturbance induced in PCa rats along the hypothalamic–pituitary–testis axis. *Spirulina* revealed a significant reduction in the level of total and free prostatic specific antigen (PSA) to the same level as bicalutamide treatment, which was the same as the control group. Histopathological study revealed regression (75%) of the histological pattern of high-grade prostatic intraepithelial neoplasia (HGPIN) for *Spirulina* alone, and (50%) for bicalutamide. The best effect on IL-6 decline was reached with the AgNPs/*Spirulina* combination as well as bicalutamide treatment compared with the PCa group. Bicalutamide treatment significantly decreased the PSA concentration relative to the PCa group and reached the normal level. Adding *Spirulina* to AgNPs as a combination enhanced its effect on all mentioned drawbacks associated with PCa except hormonal imbalance that needs more adjustments.

## Introduction

Cancer is exacerbated by increasing risk factors associated with a growing economy and globalization. In men, the prostate tumor is the most diagnosed cancer in 112 countries, and the second leading cause of death in men within 48 countries, in part because of its high frequent fatality rate. Several factors that have been established to explain these remarkable variations are related to family history of this malignancy, advancing age, definitive genetic mutations (e.g., BRCA1 and BRCA2), and conditions such as Lynch syndrome [1]. Lifestyle and dietary factors may play central roles with androgen hormones considered intermediaries between exogenous effectors and molecular sites in the development and progression of prostate cancer [2].

Silver nanoparticles (AgNPs) are considered potential anticancer materials. Many cytotoxicity studies of AgNPs have been documented. The therapeutic action of AgNPs may appear through manipulation of their shape, size, charge, elemental composition, and surface area modification or functionalization that loads target particles to specific tissues [3]. AgNPs were also screened for anticancer potential comparable to known anticancer drugs and were found to be not significantly detrimental to the normal cell line. Therefore, such synthesized AgNPs may be explored as anticancer agents [4]. The application of AgNPs can be selectively used to treat cancerous cells/tissues with no side effects, and subsequently, they may kill prostate cancer cells [5].

*Spirulina* is a filamentous multicellular blue‒green‒microalgae known as “cyanobacteria” belonging to 2 separate genera, *Spirulina* and *Arthrospira,* with approximately 15 species [6]. The basic biochemical ingredients of *Spirulina* are high amounts of protein of approximately 55–70% per dry weight, high concentrations of polyunsaturated fatty acids (PUFAs) of 1.5 to 2.0% of 5–6% total lipids, vitamins, minerals, trace elements, and many photosynthetic pigment complexes. *Spirulina* became very interesting in the research of the prevention and management of cancer. In vivo and in vitro experimental trials have proven that *Spirulina* is effective in treating cancer, inflammatory processes, and many other diseases. Several of these experiments are due to *Spirulina* itself or some of its ingredients [7].

Cancer treatment traditionally based on a single treatment is usually associated with drug resistance, limited therapeutic efficacy, and unnecessary side effects. The synergistic effect of combination therapy using two or more therapeutic agents is an alternative strategy for effective cancer management [8]. The use of drug combinations that act by a variety of pathways involved in prostate cancer (PCa) progression exhibits an alternative to remove the resistance caused by monotherapy and improve the efficacy of traditional drugs. Thus, our research aimed to study the effect of AgNPs and *Spirulina* algae for the management of PCa once as monotherapy or as a combination [9].

## Materials and Methods

### Experimental Animals

This study was performed in line with the principles of the Declaration of Ethical Committee for Institutional Animal Use and Care of the College of Veterinary Medicine, Banha University (BUFVTM-01–05- 2021). Male albino rats with a total number of 72 were used in this experiment with a recorded weight range from 250 to 330 g and an average of 268 g. Animals were housed in separate metal cages supplied with fresh and clean tap water ad libitum. They were kept under constant nutritional and environmental conditions throughout the experimental period. The animals were housed for 15 days for acclimatization before starting the experiment. All animals were fed a frequently basal ration throughout the experiment as a standard pellet diet.

### Silver Nanoparticle Preparation

The AgNPs were purchased from the local scientific agency specializing in the preparation and characterization of AgNPs. Silver nitrate and trisodium citrate were used as precursor substances for the synthesis of AgNPs. The silver colloid was prepared by using the coprecipitation method. Indefinitely, 50 ml of 0.001 M AgNO3 was heated to boil and then added to this solution 5 ml of 1% trisodium citrate drop by drop. During the process, the solutions were vigorously mixed and heated until the color changed to pale yellow. This solution was removed from the heating instrument and stirred until cooled to room temperature and preserved in the dark [10]. The suspension was preserved according to the manufacturer’s instructions and administered at a dose of 0.3 mg Ag/kg b.w/day. Each rat in the AgNPs and combination groups were given 0.5 ml from the suspension by oral gavage [11].

### Spirulina Preparation

**Fig. 1:**
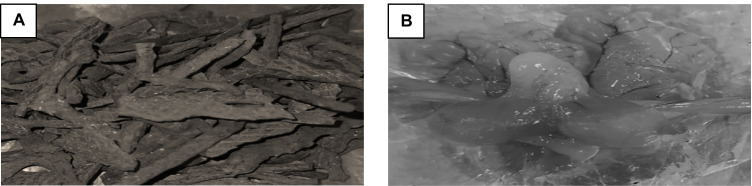
**A** Photograph of raw *Spirulina* algae rods, **B** Photograph of rat dissected urogenital tract

*Spirulina* rods were obtained from the Arab Academy for Science, as shown in Fig. 1A. The *Spirulina* suspension was freshly prepared every day by adding the raw powder to warm distilled water with stirring for 10 min until full dispersion. The suspension was administered at a dose of 4 gm/kg b.w. Each rat in the *Spirulina* group and AgNPs/*Spirulina* group was given 1 ml from the suspension by oral gavage every day.

### Silver Nanoparticle Characterization

A transmission electronic microscopy (TEM) (model EM-2100 High-Resolution- Japan) was used for microscopic class at a magnification ratio of 25 × and voltage of 200 kV, and the technique was carried out to confirm the 2D shape and size. In addition, a scanning electronic microscope (SEM) was used (model: Jol 2000, Japan). This technique was carried out for 2D shape confirmation [3].

### The Technique of the Experimental Model for Prostate Cancer Induction

We selected one of the successful attempts to induce more frequent and relevant prostate cancer in rats with combined therapy of androgenic manipulation in conjunction with a carcinogen agent. The anti-androgen bicalutamide (Casodex®) was used as the initial 3-week treatment in this experiment, and each tablet was freshly grinded and dissolved in pure dist. water and then administered orally at a dose of 15 mg/kg b.w to all groups except control animals were given pure dist. water only. This step guaranteed the shrinking of the prostate gland. Then, the rats were treated with an androgen analog, testosterone propionate (Testonon). Each ampoule (1 ml) contained 30 mg of testosterone propionate, 60 mg of testosterone phenylpropionate, 60 mg of testosterone isocaproate, and 100 mg of testosterone decanoate (eq. to a total of 176 mg of testosterone). The ampoule contents were dissolved in pure corn oil and then injected s.c. for 3 days at a dose of testosterone (100 mg/kg b.w.). Animal groups were administered testosterone, except the normal control group, which was injected with pure corn oil s.c. only. This step was guaranteed to stimulate prostate tissue regrowth.

The carcinogen 7, 12-dimethylbenz(a)anthracene (DMBA) was dissolved in physiological saline on the day after the third testosterone injection. Each rat was injected intraperitoneally during the regrowth stage at a dose of 30 mg/kg b.w. except for rats in the control group that received saline i.p. inj. only, and then testosterone was injected s.c. at an approximate dose of 2 mg/kg for 3 months. Thus, testosterone is a strong tumor promoter for the rat prostate [12–14].

### Design of the Experiment

The 72 albino rats were randomly divided into six main groups as follows:

- Group I (co): Twelve rats acted as the negative control group and were fed a basal ration in the form of a standard pellet diet, with free access to tap water during the period of the experiment (no cancer induction, no treatment)
- Group II (PCa): There were twelve rats used as a prostate cancer model according to the procedures mentioned above (no treatment)
- Group III (AgNPs): There were twelve rats with induced prostate cancer that were treated orally with silver nanoparticles at a dose of 0.3 mg/kg b.w daily for 28 days.
- Group IV (Sp): Contains twelve rats with induced prostate cancer and then treated with *Spirulina* extract at a dose of 4 g/kg b.w daily.
- Group V (AgNPs/Sp): Contains twelve rats with induced prostate cancer and then treated with silver nanoparticles at a dose of 0.3 mg/kg b.w plus *Spirulina* extract at dose 4 g/kg b.w daily.
- Group VI (a.a): Contains twelve rats with induced prostate cancer and then treated with the anti-androgen, bicalutamide (Casodex®), at a dose of 15 mg/kg b.w daily.

### Blood Samples

Rats were fasted overnight 28 days after starting the treatment. In the early morning, blood samples were collected from the medial canthus of the rat eye by ocular vein puncture. The collected blood samples were allowed to coagulate at room temperature for 30 min and then centrifuged at 3000 rpm for 10 min. The clear sera were aspirated carefully and transferred into labeled tubes and left frozen until estimation of the prostate cancer tumor markers (total PSA, free PSA, PAP), hormones (FSH, LH, E2, T), and IL-6.

### Tissue Samples for Histological Studies

After 28 days, all rats were sacrificed on the day following the final administration of treatments. The abdominal cavity was opened with a ventral midline incision from the urethera to the jaw. The urogenital tract was dissected and the anus, penis, prostate, preputial glands, vas deferens, urinary bladder, and seminal vesicles were removed in one piece as shown in Fig. 1B. Then, the prostate gland was extirpated and fixed in 10% neutral buffered formalin and instantly processed by the paraffin technique. Hematoxylin and eosin (H&E) sections were trimmed and stained for histological study [15].

### Quantification of Prostate-Specific Antigen (PSA)

The concentrations of total and free PSA in serum were evaluated by commercial ST AIA-PACK PSAII, and ST AIA-PACK free PSA test kits (TOSOH India Pvt. Ltd.) according to the manufacturer’s instructions using a TOSOH automated immunoassay analyzer.

### Prostatic Acid Phosphatase Activity Determination (PAP)

Prostatic acid phosphatase activity was determined according to Seiler and Nagel [16] using α-naphthyl phosphate as substrate. The Acid Phosphatase Assay Kit is a high sensitivity, simple, direct, and HTS-ready colorimetric assay designed to measure acid phosphatase activity in serum and other samples.

### Serum Hormone Measurements

Serum hormones FSH, LH, estradiol (E2), and testosterone (T) were measured with commercial ELISA kits (TOSOH India Pvt. Ltd.): ST AIA-PACK FSH, ST AIA-PACK LH II, ST AIA-PACK iE2, and ST AIA-PACK Testosterone respectively, according to the company’s instructions using a TOSOH automated immunoassay analyzer.

### Serum Analysis of IL-6 Cytokines

IL-6 cytokine levels were assessed in serum samples from the rats and were measured using ELISA method-based (Quantikine® ELISA) immunoassay biomarkers. The assay was performed following the manufacturer’s instructions using a Ryto 2100c microplate reader.

### Statistical Analysis

First, all results were tested for normality and homogeneity patterns. Then, one-way ANOVA was used to determine the statistical significance of differences for all groups followed by a post hoc test (Duncan’s test) for making multiple comparisons using the Statistical Package for Social Science Software (Version 25, SPSS Inc., Chicago, IL, USA). The values are represented as the mean ± standard error of the mean. A significant difference was used =  < 0.05 probability level.

## Results

### Silver Nanoparticle Characterization by SEM and TEM Techniques

**Fig. 2:**
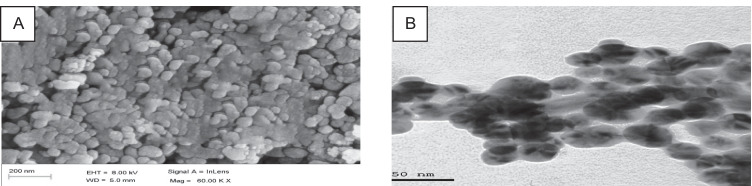
Images of silver nanoparticle characterization, **A** Photograph of SEM image, **B** Photograph of TEM image

Figure 2 shows a 2D TEM image and a 3D SEM image for silver. In the SEM images, the spherical shape of AgNPs is illustrated with homogenous shape and size as shown in Fig. 2A. Additionally, the TEM image (Fig. 2B) illustrated a spherical shape to subspherical, but the dominant shape was spherical with a size of approximately 65 nm.

### Changes in Tumor Markers (serum total PSA, free PSA, and PAP)

**Fig. 3:**
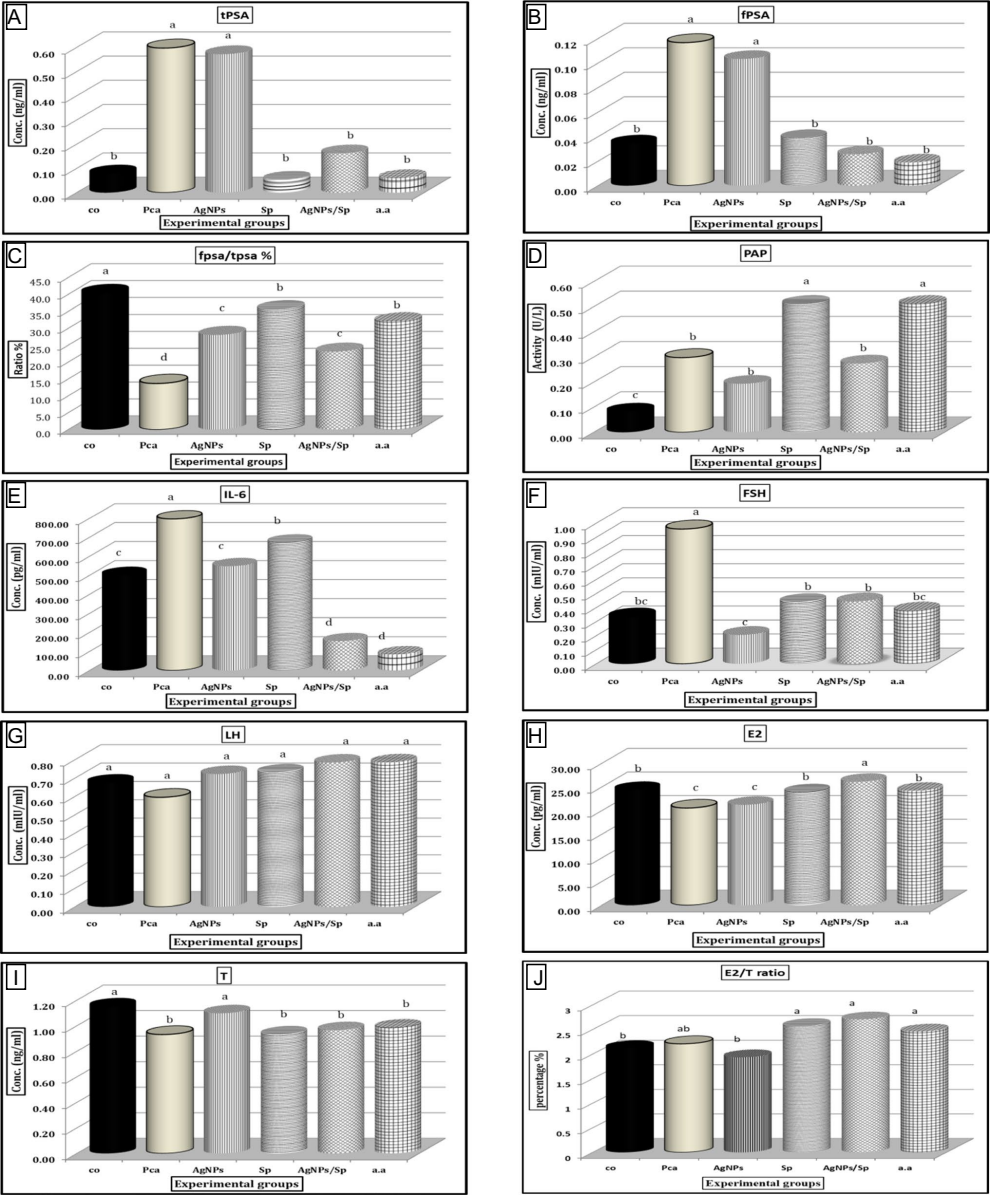
Effects of treatments on serum biochemical parameters of experimental groups, Co: control, PCa: prostate cancer, AgNPs: silver nanoparticles, Sp: *Spirulina*, AgNPs/Sp: combination treatment of silver nanoparticles, and *Spirulina*, a.a: anti-androgen, **A** Total prostatic specific antigen, **B** Free prostatic specific antigen, **C** Free PSA/total PSA ratio, **D** Prostatic acid phosphatase, **E** Interleukin-6, **F** Follicular stimulating hormone, **G** Luteinizing hormone, **H** Estradiol, **I** Testosterone, **J** E2/T ratio, (a, b, c, d, and e) there is no significant difference (*P* > 0.05) between any two means that have the same superscript letter

The findings on serum total PSA and free PSA in the study groups are described in Fig. 3. The adenocarcinoma prostate model group showed extremely significant increases of more than 7- and fourfold in the mean total PSA and free PSA concentrations, respectively relative to the negative control group. After treatment with AgNPs, the concentrations of total PSA and free PSA were still significantly higher than those in the control group and at the same level as the PCa group, but the treatment groups with *Spirulina* alone, combined (AgNPs/Sp), or bicalutamide showed a significant decrease in the mean PSA concentration relative to the PCa group and reached the normal level as the control group. The ratio of total PSA and free PSA significantly declined in the PCa group compared with all other groups; hence, a high ratio was observed in the control group. Each of the *Spirulina* and bicalutamide groups had the same total PSA and free PSA ratio, which was significantly higher than that of the PCa group and AgNPs and combined (AgNPs/Sp) groups.

Prostatic acid phosphatase activity was significantly higher in the *Spirulina* and bicalutamide groups, while the AgNPs and combination groups had nonsignificant levels compared with the PCa group. It is noticed that each *Spirulina* and bicalutamide group contributed to elevating the PAP activity over the PCa. There was a slight decrease in PAP activity in the AgNPs, and combination groups compared with the PCa group, although the difference was not statistically significant.

### Changes in Serum Hormone Concentrations (FSH, LH, E2, and T)

The prostate cancer group showed a very high level of FSH, approximately threefold the level of the negative control group. After treatment with *Spirulina*, combination, or bicalutamide, the level of FSH reached the normal level as in the control group.

Silver nanoparticles could significantly decrease the level of FSH relative to all other experimental groups, even the negative control group. AgNPs have the same effect on FSH levels as bicalutamide since each of them could achieve the lowest FSH level.

Changes in luteinizing hormone did not reveal significant differences between all experimental groups. Although statistically nonsignificant, the bicalutamide group showed the lowest LH level followed by the combination group.

The estradiol level dropped down in the prostate cancer group, while each of the *Spirulina* and bicalutamide treatments normalized the E2 level compared with the control group. Combination treatment significantly increased the E2 level relative to all other groups, while AgNP treatment achieved the lowest effect which is similar to the PCa group.

There was a significant decline in testosterone concentration in the PCa group, but AgNPs could overcome this decline to the normal level observed in the control group. The other groups could not make a difference in serum testosterone concentration compared with the PCa group.

### Changes in Serum IL-6 Levels

The lowest level of interleukin-6 was seen in the combination and bicalutamide groups while the highest level was in the PCa group compared with the negative control group. AgNP treatment normalized the IL-6 level to the control group level. *Spirulina* treatment alone significantly decreased the IL-6 level compared with that in the PCa group but was still higher than that in the control group.

### Histopathological Findings

**Fig. 4:**
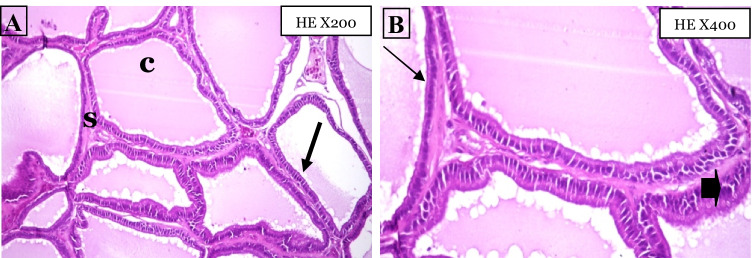
Photomicrographs cross-sections of the prostate gland for the negative control group, **c**: luminal prostatic concretions, **S:** thin strip of interglandular smooth muscle fibers, **(thick arrow)**: thin intraglandular epithelia lining, **(thin arrow)**: basal epithelial cells, **(arrowhead)**: secretory normal epithelial cells

**Fig. 5:**
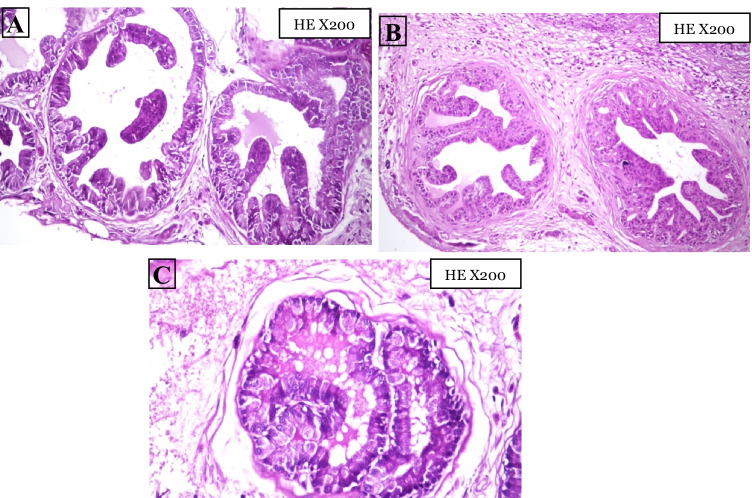
Photomicrographs of cross-sections of the prostate gland for the cancer group, **A** micropapillary, **B** tufted, and **C** cribriform, tufted (HE X200)

The histopathological analysis in Fig. 4 shows that the negative control group has normal prostatic acini with thin strips of interglandular smooth muscle fibers, thin intraglandular epithelial lining, and luminal prostatic concretions. The thin intraglandular epithelial lining is cuboidal, columnar, or transitional with two types of cells: basal epithelial cells and secretory normal epithelial cells. Photomicrographs of the cross-section of the cancer group prostate gland shown in Fig. 5 have proven the success of the.

current method, materials, and duration used in this in vivo experiment to induce prostate adenocarcinoma in rats. The PCa group had high-grade prostatic intraepithelial neoplasia (HGPIN) which is an abnormal prostate gland and is believed to precede the development of prostate adenocarcinoma (the most common form of prostate cancer). It may be simply referred to as prostatic intraepithelial neoplasia (PIN). It is considered a premalignancy or carcinoma in situ of the prostate. HGPIN is histologically characterized here by preexisting (noninvasive) ducts and acini usually medium to large in size and lined by crowded cells with abnormal cryptologic features have 4 major architectural patterns: flat, micropapillary, tufted, and cribriform.

**Fig. 6:**
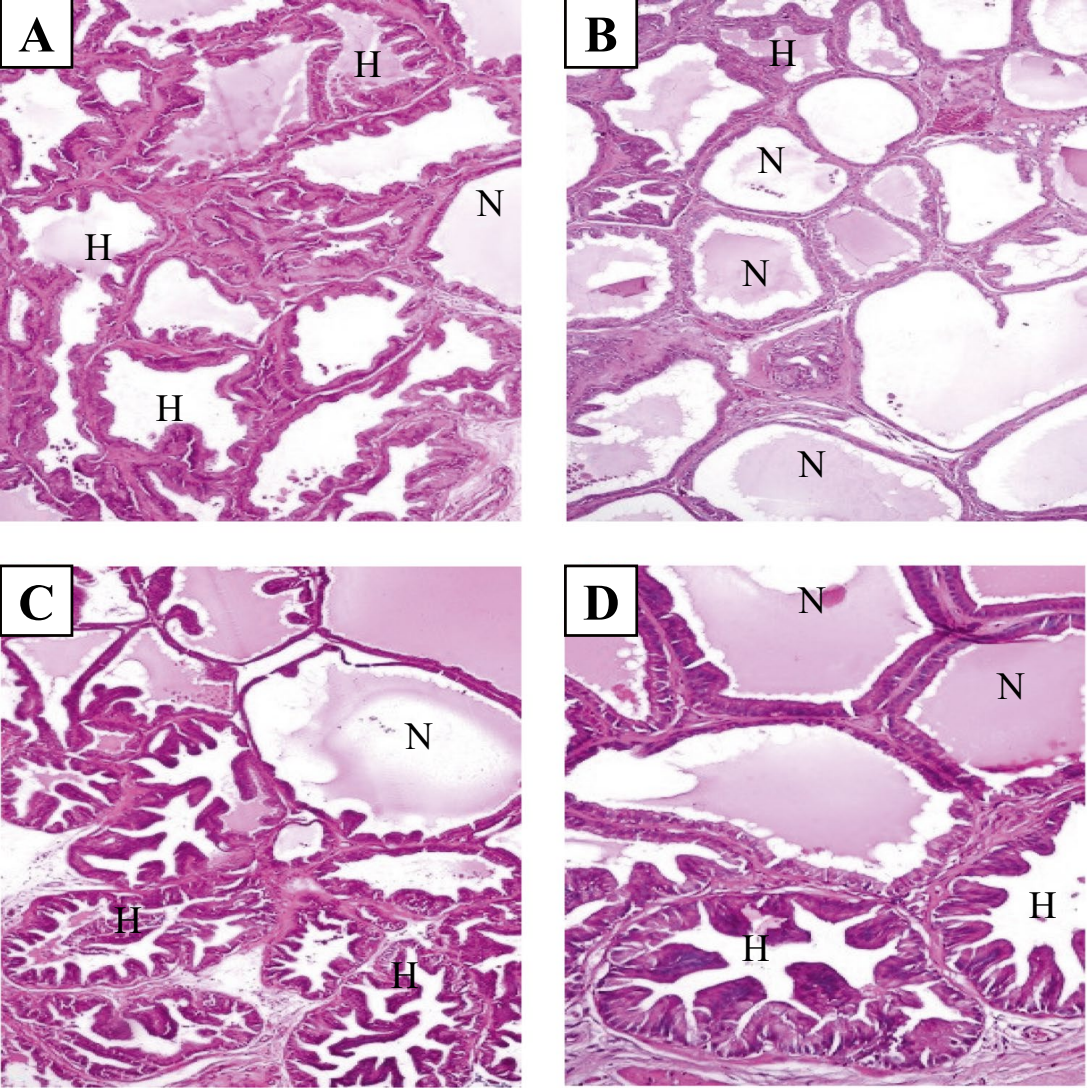
Photomicrograph cross-sections of the prostate gland for treated groups, **H** High-grade prostatic intraepithelial neoplasia, **N** normal prostatic acini, **A** AgNPs group, **B** Spirulina group, **C** combination AgNPs/Sp group, **D** a.a group (HE X200) indicating regression of the histological pattern of HGPIN, which replaced by normal prostatic acini with different percent among each group with approximately 10, 75, 25, and 50% for silver nanoparticles, Spirulina, combination, and bicalutamide groups, respectively

Photomicrographs of cross-sections of the four treatment groups (Fig. 6) indicated regression of the histological pattern of high-grade prostatic intraepithelial neoplasia, which was replaced by normal prostatic acini with different percentages among each group with approximately 10, 75, 25, and 50% for silver nanoparticles, *Spirulina*, combination, and bicalutamide groups, respectively.

## Discussion

The prostate cancer model has been correctly established by a preparation step (anti-androgen, then testosterone administration) followed by chronic treatment with testosterone after administration of carcinogens (DMBA). Testosterone significantly enhances prostate cancer even at doses that do not remarkably increase serum testosterone; thus, testosterone acts as a strong tumor promoter in the rat prostate. After the testosterone injection period, the treatment period started and continued for 28 days. During this time, the exogenous hormone supply was stopped, so the prostate gland was dependent on the remaining level of testosterone, in addition to normal original sources, which started to compensate for the action of exogenous testosterone withdrawal [17, 18].

In male serum, PSA is present as a complex with α1-antichymotrypsin (PSA-ACT) and α2-macroglobulin (PSA-A2M), and as free PSA [19, 20]. PSA was more sensitive than PAP in the serum detection and screening of prostate cancer. However, the use of PSA leads to overdiagnosis and overtreatment of prostate cancer. Moreover, it is not effective in the prediction of metastases and prognosis, especially prognosis after surgery. High-grade prostatic intraepithelial neoplasia (HGPIN) occurring in rats is being approved by elevating tumor markers total PSA, free PSA, PAP, and pathological findings [21].

AgNP treatment alone did not improve the PSA tumor marker. Each of the AgNP and combination treatments had no effect on the PAP level because the level was still high as in the PCa group relative to the control group. These results are concurrent with those of **Sheida et al. (2021),** who mentioned morphological changes, and some marker changes have been found in different nanoparticles in the prostate. For example after zinc nanoparticles and nanosized titanium dioxide treatment, PSA and PAP concentrations were increased [22].

The ratio of free PSA/total PSA significantly declined in the PCa group compared with all other groups, which is consistent with a clinical study by **Etzioni et al. (2004), and Catalona et al. (1998),** who found that serum PSA in PCa cases increase and free PSA decreases in comparison to patients with benign prostatic hyperplasia; hence, a high ratio was seen in the control group [23, 24]. Each of the *Spirulina* and bicalutamide groups, similar to the AgNP and combined (AgNPs/Sp) groups had the same fPSA/tPSA ratio, which was significantly higher than that of the PCa group.

IL6/IL6RA signaling plays an important role in the progression of various cancers including prostate cancers. The levels are increased under these conditions [4]. It is a multifunctional cytokine that modulates the differentiation and proliferation of cancer cells in both an autocrine and paracrine manner [25]. AgNPs normalized the level of IL-6 to levels similar to those in the control group and significantly lower than the *Spirulina* group compared with those in the PCa group. Similar results were reported by **Carlson et al. (2008),** who revealed that after 24 h of exposure of culture media to silver nanoparticles (15 nm), a significant inflammatory response but no detectable level of interleukin-6 was observed [26]. Furthermore, **Muniyappan and Nagarajan (2014)** concluded that silver nanoparticles exhibited considerable antioxidant and anti-inflammatory activity [27]. In contrast, **Ramadi et al. (2016)** found that approximately 24 h after the induction of AgNPs, a dose-related increase was observed in the upregulation of the expression of several pro-inflammatory gene mediators [28]. Additionally, concerning the IL-6 receptor, **Singh et al. (2016)** demonstrated that AgNPs increased IL6RA secretion production in a cell line [4].

Elevated levels of FSH are associated with unresponsive gonads or hyperfunctioning pituitary adenomas. In addition, the prostate gland itself synthesizes FSH and expresses FSH receptors in pathologic states (BPH and prostate cancer) [29]. Here, the PCa group showed a tremendous elevation in FSH levels that reached more than threefold the normal level, which is emphasized in a clinical study by **Heracek et al. (2007)** [30] for FSH levels and by **Mariani et al. (2006)** [31] for the FSH receptors.

AgNPs normalized the level of FSH compared with that in the PCa group, similar to the effect of bicalutamide, and removed the pathologic elevation that was accompanied by a cancerous state. These results agreed with **Olugbodi et al. (2020),** who found significant dosage-dependent decreases in follicle-stimulating hormone (FSH) in rats treated with AgNPs for 7 and 28 days [32]. However, others registered nonsignificant changes in different treated groups during the whole period of the experiment when compared with the control group [33–35]. The deviation in FSH results is due to cytotoxic research performed on normal rats not on pathological models.

Luteinizing hormone (LH) is a pituitary hormone that is considered essential for sexual development and reproduction in men and women. LH is governed by gonadotropin-releasing hormone (GnRH) from the hypothalamus which is sensitive to circulating levels of sex hormones. Elevated serum levels of both LH and testosterone may increase the risk of prostate cancer. In the present study, the level of LH was not significantly different among all groups. This may be because its level is influenced by pulsatile secretion and diurnal variation, which complicates the assessment of their roles in the prostate [36]. Although many studies have shown an increase in LH levels accompanied by a decrease in testosterone concentrations upon AgNP treatment [33–35]. One study demonstrated the opposite effect [37]. However, none of these studies were performed on a rat PCa model, as we have done [38].

AgNPs significantly increased testosterone levels to normal levels, and kept E2 levels unchanged compared with PCa rats moreover, the E2/T ratio was normal, similar to the control group. **Ajayi et al. (2021)** obtained the same results when using biogenic AgNPs to treat hormone-induced prostate enlargement, which has been ameliorated compared to placebo and significantly increased serum testosterone levels [39]. Overall, AgNPs could overcome hormonal disturbance induced in PCa rats along the hypothalamic–pituitary–testis axis.

*Spirulina* reduced the levels of total PSA and free PSA to the same level as bicalutamide treatment, which was statistically the same as the negative control group. Each of the *Spirulina* and bicalutamide groups had the same free PSA/total PSA ratio, which was significantly higher than that of the PCa group, indicating PCa improvement.

The effect of *Spirulina* on the tumor markers studied here resembles the action of antiandrogen (bicalutamide) throughout the decline in PSA levels, increase in the fPSA/tPSA ratio, and markedly increase in PAP levels. The situation is different for PAP level since all groups have a significant elevation in PAP level compared to the control group, especially *Spirulina* and bicalutamide treatments could extremely significantly increase PAP level relative to control group, and relative to PCa group. According to **McCarty et al. (2014),** phycocyanobilin in *Spirulina* has the potential to blunt oxidative stress-mediated upregulation of NF-κB activity via inhibition of NADPH oxidase complexes, thereby diminishing prostate cancer AR expression and suppressing PSA production [40]. In contrast, **Kumar et al. (2009)** found that *Spirulina platensis* at 400 mg/kg orally significantly decreased serum alkaline phosphatase and acid phosphatase activities in collagen-induced arthritis (CIA) rats [41]. The opposite results were obtained in our work because we used a tenfold (4 gm/ kg) dose compared with that used in the **Kumar et al.** study.

The IL-6 level in the *Spirulina* group was decreased compared to that in the PCa group. Although this effect was lowered by *Spirulina* extract, the effect was still significantly higher compared with the control group. The effect of AgNPs was more pronounced than that of the *Spirulina* treated groups. In vivo experimental models, mouse arthritis [42], mouse-ear edema [43], mouse-paw edema [44], and rat colitis [45] support our findings; hence, phycocyanin, a biliprotein obtained from the microalgae *Spirulina* (Arthospira) maxima, exerts anti-inflammatory activity by reducing LTB4 levels, TNF-α, NO, and arachidonic acid metabolites involved in the inflammatory response [46]. But others reported the opposite direction when LPS-activated macrophages and monocytes were treated with *Spirulina* [47]. This study demonstrated that *Spirulina* had no effect on IL-6 secretion [48]. The cause for these interactions may be due to the type of model studied.

Our results revealed that FSH levels were restored to normal levels by *Spirulina* treatment similar to the bicalutamide group, compared with PCa rats which are concurrent with **Ibrahim et al. (2021),** who investigated *Spirulina platensis* supplementation with lead acetate and showed that, it could reverse the previous deteriorations. More confirmation, *Spirulina platensis* alleviated serum testosterone, FSH, and LH alterations [49].

The *Spirulina* group significantly restored E2 levels to the normal range as the bicalutamide group, but its effect on testosterone was not changed relative to the PCa group, which resulted in an elevated E2/T ratio relative to the normal group. Many researchers have demonstrated that *Spirulina* restores the production of testosterone through steroidogenesis as well as the expression levels of genes related to cholesterol transport and testosterone synthesis; however, these studies did not cover the relationship with E2 levels [50–52]. Thus, the systemic hormonal imbalance was still excited with *Spirulina* treatment, in contrast to findings for AgNP treatment. We can conclude that AgNPs can ameliorate the hormonal disturbance associated with PCa induced experimentally across all glands involved in the hypothalamic–pituitary–testis axis, but *Spirulina* extract exerts its effect locally.

The combination of AgNPs and *Spirulina* could decrease the total and free PSA to normal levels as well as the bicalutamide group, while the PAP level was significantly unchanged, resembled the AgNPs alone, and remained high over the normal range compared with PCa rats. The best effect on IL-6 decline was reached with the AgNP/*Spirulina* combination and bicalutamide treatment compared statistically with the PCa group.

The AgNP/*Spirulina* combination decreased the improper FSH levels to normal levels accompanied by no changes in LH and testosterone levels relative to the PCa model but markedly increased the E2 level which elevated the E2/T ratio over the normal range. Therefore, prostate cancer tissues in the combination group suffer from more hormonal imbalance than *Spirulina* treatment or AgNPs alone. This hormonal imbalance is due to the *Spirulina* effect, which may be due to alterations in steroidogenesis enzymes that need more investigation.

Not only these biochemical indicators have been studied, but also the histopathological study revealed regression of the histological pattern of high-grade prostatic intraepithelial neoplasia, which was replaced by normal prostatic acini when using *Spirulina* alone (75%) or in combination with AgNPs (25%). Hence, the transforming percentage of high-grade prostatic intraepithelial neoplasia by normal prostatic acini increased from 10% with AgNPs to 25% when adding *Spirulina* as a combination treatment but did not reach the bicalutamide effect (50%).

**In conclusion,** the PCa model undergoes tumor marker elevation, hormonal imbalance, inflammatory reactions, and HGPIN pathological conditions. AgNPs could improve inflammatory conditions and hormonal imbalance, while *Spirulina* could successfully decrease tumor markers and handle HGPIN pathological conditions. Introducing *Spirulina* to AgNPs in combination enhanced its effects on all mentioned drawbacks associated with PCa except hormonal imbalance, which requires more adjustments for the relative dosage between AgNPs and *Spirulina*, in addition, to study the role of enzymes involved in steroidogenesis.

## Data Availability

The datasets generated during the current study are available from the corresponding author on appropriate request.
